# Divide and Conquer (DC) BLAST: fast and easy BLAST execution within HPC environments

**DOI:** 10.7717/peerj.3486

**Published:** 2017-06-22

**Authors:** Won Cheol Yim, John C. Cushman

**Affiliations:** Department of Biochemistry and Molecular Biology, University of Nevada—Reno, Reno, NV, United States of America

**Keywords:** BLAST, Sequence similarity, Parallel processing, Environment, Distributed computing, HPC

## Abstract

Bioinformatics is currently faced with very large-scale data sets that lead to computational jobs, especially sequence similarity searches, that can take absurdly long times to run. For example, the National Center for Biotechnology Information (NCBI) Basic Local Alignment Search Tool (BLAST and BLAST+) suite, which is by far the most widely used tool for rapid similarity searching among nucleic acid or amino acid sequences, is highly central processing unit (CPU) intensive. While the BLAST suite of programs perform searches very rapidly, they have the potential to be accelerated. In recent years, distributed computing environments have become more widely accessible and used due to the increasing availability of high-performance computing (HPC) systems. Therefore, simple solutions for data parallelization are needed to expedite BLAST and other sequence analysis tools. However, existing software for parallel sequence similarity searches often requires extensive computational experience and skill on the part of the user. In order to accelerate BLAST and other sequence analysis tools, Divide and Conquer BLAST (DCBLAST) was developed to perform NCBI BLAST searches within a cluster, grid, or HPC environment by using a query sequence distribution approach. Scaling from one (1) to 256 CPU cores resulted in significant improvements in processing speed. Thus, DCBLAST dramatically accelerates the execution of BLAST searches using a simple, accessible, robust, and parallel approach. DCBLAST works across multiple nodes automatically and it overcomes the speed limitation of single-node BLAST programs. DCBLAST can be used on any HPC system, can take advantage of hundreds of nodes, and has no output limitations. This freely available tool simplifies distributed computation pipelines to facilitate the rapid discovery of sequence similarities between very large data sets.

## Introduction

Sequence-based homology searches are used widely for the analysis of nucleic acid and amino acid sequence information. However, query-based searches, such as the National Center for Biotechnology Information (NCBI) BLAST (Altschul et al., 1990) and biosequence analysis using implementations of profile hidden Markov model (HMM) methods (e.g., HMMER) (Eddy, 2011), are computationally intensive and were developed prior to the information explosion that has resulted from next-generation sequencing (NGS) technologies. Timely processing of massive NGS data often require data parallelization. BLAST+ improves BLAST speeds by breaking long sequences into shorter ones for processing and leveraging the multicore architecture of modern microprocessors (Camacho et al., 2009). Alternatively, the parallel local alignment search tool (PLAST) exploits multithreading targeting multicore (2–8 cores) and many-core (dozens to hundreds of cores) architectures and single instruction multiple data (SIMD) parallelism using multicore microprocessors to speed up the processing of large datasets by three- to six-fold (Nguyen & Lavenier, 2009). However, these parallelization programs use multicore or multithread approaches within a single computer or node and report Expected *(E)*-values that differ from those obtained using the NCBI BLAST+ algorithm.

The recent emergence of high-performance computing clusters and distributed grid and cloud computing resources (Foster, 2003; Foster et al., 2008) and graphics processing units (GPUs) (Owens et al., 2008) have significantly reduced the run times of bioinformatics software. Cluster, grid or HPC environments with multiple nodes provide large computational capacities that can significantly accelerate program execution speeds through efficient job scheduling and parallelization across multiple nodes. Also, GPUs have massively parallel programming unit architectures within a single hardware unit, which allows them to perform more robustly than single-CPU processors (Owens et al., 2008). However, developers must apply specialized single-instruction multiple thread (SIMT) programming skills in order to take advantage of the massively parallel programming unit architectures of GPU cores (Owens et al., 2008; Vouzis & Sahinidis, 2011).

A number of parallel BLAST applications have been developed, including GridBLAST (Krishnan, 2005), CloudBLAST (Matsunaga, Tsugawa & Fortes, 2008), mpiBLAST (Lin et al., 2011), HPC-BLAST (Sawyer et al., 2015), PLAST (Nguyen & Lavenier, 2009), ScalaBLAST (Oehmen & Baxter, 2013), a GPU-based BLAST (Ling & Benkrid, 2010), GPU-BLAST (Vouzis & Sahinidis, 2011), and SCBI_MapReduce (Guerrero-Fernández, Falgueras & Claros, 2013). While these applications improve the execution time of BLAST, their compilation and configuration are complicated to varying degrees depending up on the libraries and platforms used.

Here, Divide and Conquer BLAST (DCBLAST) is introduced as a rapid and easy-to-use implementation wrapper for NCBI BLAST+ that enables execution of sequence alignment-based searches in cluster, grid, or HPC environment using a simple command-line interface. DCBLAST operates by automatically dividing the query sequences into a user-defined number of subsequences (*N*), submitting the distributed job to the computing environment, and then merging the returned NCBI BLAST+ results. This approach dramatically reduces job run times and is amenable to all large-scale BLAST or BLAST+ analyses. This method allows the processor to obtain each job independently and to ensure that each job has equivalent query sizes. This query size balancing is critical because BLAST search execution time depends upon the length of the sequence, not upon the number of sequences (Matsunaga, Tsugawa & Fortes, 2008; Oehmen & Baxter, 2013). Because DCBLAST can use as many CPUs as are available within a particular cluster or HPC, improvements in its BLAST performance are dependent upon the number of CPUs available. Although only experiments with BLAST are described here, the proposed solution can also be applied to other applications with similar execution profiles, such as HMMER (Eddy, 2011). We demonstrate the performance of DCBLAST using a high-performance computing system that has 27 high-end nodes with 16 core processors each.

## Materials and Methods

**Figure 1:**
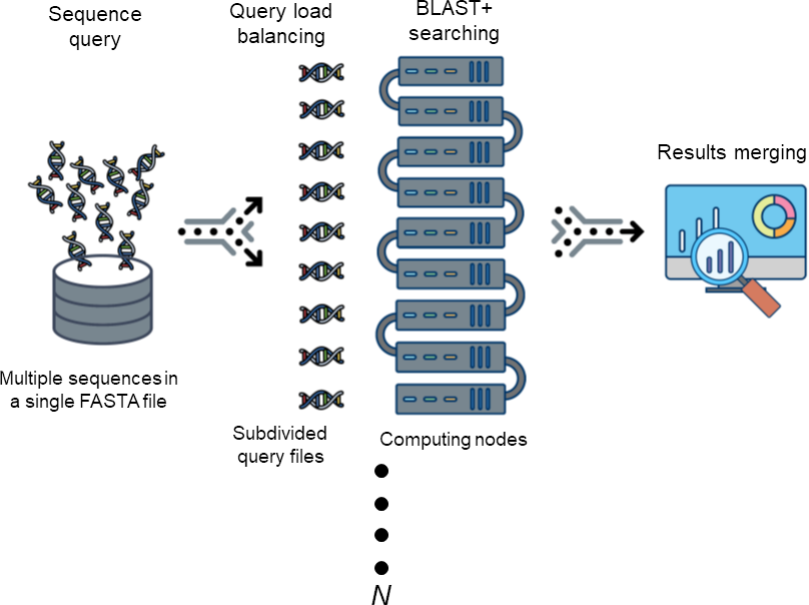
The workflow of DCBLAST. Sequence query involves submission of a single FASTA file containing multiple sequences. The sequences are then subdivided into multiple sequence query FASTA files to achieve load balancing across multiple computer nodes. After BLAST/BLAST+ searching has been completed, the results from each search are merged into a single output file.

**Description.** DCBLAST is an NCBI BLAST/BLAST+ wrapper that enables straightforward parallelization and is available as open source. Regardless of the number of sequences, the BLAST job run time is affected by the total sequence length of a query. Thus, DCBLAST applies query size balancing by dividing input sequences into equivalently sized files. This technique reflects a distributed computing approach that uses the functional programming philosophy of MapReduce (Dean & Ghemawat, 2008). DCBLAST was constructed using the Perl scripting language (http://www.perl.org) and additional modules can be downloaded to support the development of DCBLAST including Config::Tiny, Data::Dumper, and Path::Tiny (http://www.cpan.org). DCBLAST runs on Linux- or Unix-based systems, which allow jobs to be submitted through the Sun Grid Engine (SGE) to process parallelized tasks. DCBLAST can be implemented using any version of NCBI BLAST+ (Camacho et al., 2009). While NCBI BLAST+ settings can affect the results, any desired configuration of non-default values can be provided by the user. Also, DCBLAST supports all NCBI BLAST/BLAST+ suite variations including BLASTN, BLASTX, BLASTP, TBLASTN, TBLASTX, and PSI-BLAST. Alternatively, the user can alter each option according to their particular HPC system and needs. DCBLAST operates by automatically dividing the query sequences into a user-defined number of subsequences (*N*), submitting the distributed job to the computing environment, and then merging the NCBI BLAST+ alignment results. An overview of these process steps is illustrated in Fig. 1.

**Figure 2:**
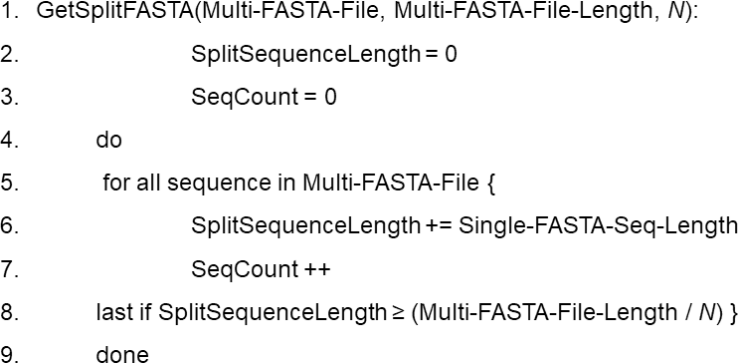
Pseudocode for the DCBLAST algorithm to perform query subdividing. The multiple query sequences (in one FASTA file) are then subdivided into a set of query files until the subdivided lengths exceed that of the total length/*N*. Once subdivided, the program will then submit the individual subdivided files to the HPC scheduler and BLAST/BLAST+ is carried out. Lastly, the BLAST/BLAST+ output files are merged into a single report file that is returned to the user.

**Data preprocessing.** DCBLAST executes query subdivision to ensure that all the jobs have more or less equivalent query sizes, and that the number of query subsequences (*N*) is based upon the user-defined number of CPUs available for a particular job according to queue status. Multi-FASTA format files are used to provide an initial estimate of the total sequence length within the query file and these Multi-FASTA files are then divided by *N*. This produces a query comprised of minimal and approximately equal-sized subdivided FASTA files. The use of approximately equal-sized queries, instead of an equivalent number of sequences, maximizes CPU utilization and facilitates more rapid and balanced processing times. This balancing approach is critical because BLAST search execution time depends upon the length of the sequence, not the number of sequences (Matsunaga, Tsugawa & Fortes, 2008). This step is the key aspect of DCBLAST that reduces computational time. DCBLAST pseudocode is presented in Fig. 2.

**Job submission.** After query subdivision, DCBLAST automatically passes the query subsequences to the job submission processor. DCBLAST can run a user-defined number of jobs (*N*), if sufficient numbers of CPUs are available within a particular cluster, grid, or HPC environment. The performance improvements obtained by DCBLAST are dependent upon the number of CPUs. The Sun Grid Engine (SGE) can be used for job queuing on the master node. DCBLAST can handle the job submissions as an array. The SGE supports the concept of an array job, which is submitted to the cluster once and can be managed by a single job ID, rather than having to manage thousands of independent job IDs. DCBLAST sets a variable called ‘SGE_TASK_ID’, which is based upon the number of query subsequences, and which can be used within the job script to identify the correct query file to be used for each job task.

**Output.** Standard output and standard errors are generated for array jobs submitted as serial jobs. Upon job completion, DCBLAST generates a log file with an error log that includes specific query names and error codes for each task. If no errors occur, then the file remains empty. Also, the error log indicates whether an erroneous query file was used, so that the user can check it and run the correct query file, rather than running the entire DCBLAST job again. When complete, DCBLAST creates an output file in the ‘results’ output directory. DCBLAST merges all array jobs results into a single file, and this result file is formatted using standard NCBI BLAST/BLAST+ parameters.

## Results and Discussion

Proof-of-concept and benchmarking experiments were conducted in order to determine the relative efficiency of DCBLAST. The demonstration was done on the University of Nevada (UNR) high-performance computing (HPC) cluster (nicknamed the ‘Grid’) using 27 high-end nodes with a dual eight (16)-core, 2.6 GHz CPU (Intel^®^ Xeon^®^ Dual E5-2650 v2) processors and 256 Gb RAM that are managed by the SGE (http://www.unr.edu/it/research-resources/the-grid). DCBLAST was tested using 1, 8, 16, 32, 64, 128, and 256 CPU cores across HPC nodes. The benchmark times included subdivision of sequence queries, job submission, completion of BLAST searches, and merging of the results.

**Figure 3:**
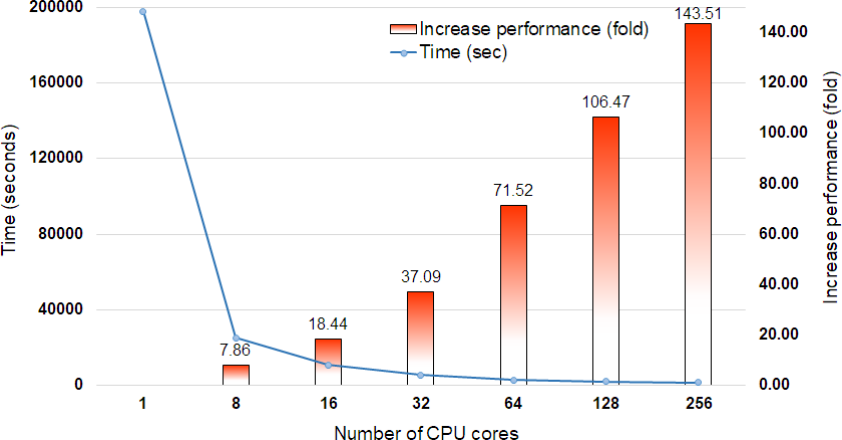
Scaling performance of DCBLAST with *Arabidopsis* query transcripts *versus* the UniProtKB/Swiss-Prot protein database. Speed benchmarks shown include processing times in seconds and fold increases in performance when using 1, 8, 16, 32, 64, 128, and 256 CPU cores.

DCBLAST performance metrics were collected after running a query of 35,386 *Arabidopsis* transcripts from The Arabidopsis Information Resource (TAIR) 10 database (Lamesch et al., 2012) containing 43,546,761 bp on the UNR HPC cluster. All query sequences were compared against the UniProtKB/Swiss-Prot protein database (release-2014_07) (The UniProt Consortium, 2013) using the BLASTX option with max sequence 1, *E*-value cut off 1-e10, and print option 9. The UniProtKB/Swiss-Prot database (release-2014_07) contained 546,000 proteins represented by 194,259,968 amino acid sequences. Scaling performance of DCBLAST is shown in Fig. 3. DCBLAST showed accelerated execution times that increased nonlinearly with an increasing number of CPU cores. Scaling from one CPU core to 256 CPU cores resulted in more than a 143.51-fold improvement in processing speed. However, there is not a clear correlation between the execution time and the number of CPU cores because the run also includes the splitting of the query sequences and merging of the results. DCBLAST displayed similar performance characteristics for all programs within the NCBI suite of BLAST/BLAST+ programs and generated identical *E*-value scores. Lastly, DCBLAST serves as an alternative solution to NCBI BLAST+ search for faster analysis while guaranteeing identical results.

In order to showcase the relative utility of DCBLAST, we have summarized its features in comparison to a set of widely used and parallelized variations of BLAST (Table 1). First, to make DCBLAST easy to use, we sought to avoid cumbersome prerequisites such as the need to create complex file structures associated with the database. However, the same is not true for a number of other parallel BLAST programs that have been released that use libraries such as Hadoop, Message Passing Interface (MPI), Open Message Passing (OpenMP), and Remote Procedure Call (RPC) to facilitate distributed programming (Gropp & Lusk, 1997; Dagum & Menon, 1998; Sato, Boku & Takahashi, 2003; Shvachko et al., 2010). Typically, these distributed programs are difficult to compile because many prerequisite libraries must be installed. For example, an MPI-based approach requires a specific MPI library style (e.g., MPICH, IntelMPI, BlueGene, MVAPICH, OpenMPI, or other styles), that requires a particular type of network protocol and operating system. Although mpiBLAST is the most highly recommended method for performing large-scale sequence similarity searches (Lin et al., 2011), it does not currently support NCBI-BLAST+ and has limited output formatting options (Table 1).

**Table 1:** Comparison of the features of DCBLAST with those of existing parallel bioinformatics software for the performance of BLAST/BLAST+ searches.

Features	DCBLAST	HPC-BLAST	GPU-BLAST	PLAST	mpiBLAST	ScalaBLAST
Parallelize algorithm	MapReduce	MPI^a^	SIMT^b^	Ordered Index Seed	MPI	MPI
Hardware requirement	HPC^c^ environment	HPC environment (Xeon & Xeon phi)	NVIDIA^d^ GPU^e^ platform	SIMD^f^ instructions set (SSE^g^ 2+ ) supported CPU	HPC environment	HPC environment
Prerequisites	Sun Grid Engine, Perl (any version 5), Path::Tiny (Perl module), Data::Dumper (Perl module), Config::Tiny (Perl module)	Intel MPI C/C++ compiler, xild (Intel linker), xiar (Intel archiving)	CUDA^h^ 2.3+ , GCC^i^ v4.8.2+	GCC v4.4+ , cmake 2.8+	mvapich2 v1.4.1 or mvapich2 v1.4.1 2 or mvapich v1.2.0 3 or OpenMPI v1.4.1 or Intel MPI C/C++ compiler	Intel C/C++ compiler, OpenMPI
Scalable across multithreads	Yes	Yes	Yes (GPU)	Yes	Yes	No
Scalable across multinodes	Yes	Yes	Not applicable	No	Yes	Yes
Support BLAST version	All version of NCBI-BLAST+	All version of NCBI-BLAST+	Not applicable	Not applicable	NCBI-BLAST 2.2.20	NCBI-BLAST 1.1.1.1
Bibliography reference	This report	Sawyer et al. (2015)	Vouzis & Sahinidis (2011)	Nguyen & Lavenier (2009)	Lin et al. (2011)	Oehmen & Baxter (2013)
Last update	4/18/17	08/25/16	02/09/16	04/21/16	11/28/2012	08/12/13
Limitations	None	Only BLASTN and BLASTP	Only BLASTP	Use only single node/similar result to NCBI-BLAST	Limited output format/Older version of BLAST	Older version of BLAST

**Notes.**

a MPI, Message Passing Interface.

b SIMT, Single-Instruction Multiple-Thread.

c HPC, High Performance Computing.

d NVIDIA, Nvidia corporation.

e GPU, Graphics Processing Units.

f SIMD, Single Instruction Multiple Data.

g SSE, Streaming SIMD Extensions.

h CUDA, Compute Unified Device Architecture.

i GCC, GNU Compiler Collection.

The Environment Modules package (Furlani, 1991; Furlani & Osel, 1996) is a software environment that can help simplify shell initialization for various software packages, such as MPI, without requiring familiarity with the entire software package. Most compilers used in parallel computing cannot generate high-performance code without significant guidance from the developer (Bacon, Graham & Sharp, 1994; Oehmen & Baxter, 2013). In addition, HPC cluster computing environments tend to employ a heterogeneous environment and libraries supporting a wide variety of applications. Thus, their use can be challenging for the novice user. In contrast, DCBLAST is more accessible to the novice user because it does not require extensive time, effort to compile the source code, preformat queries and associated databases.

Other BLAST options, such as HPC-BLAST, provide heterogeneous and adaptive performance on any number of Xeon Phi and Xeon clusters (Sawyer et al., 2015). HPC-BLAST provides an excellent reference manual and explains the best way to compile NCBI-BLAST+ and mpiBLAST as well as HPC-BLAST. However, HPC-BLAST only supports BLASTN and BLASTP, but not the complete NCBI-BLAST+ suite (Table 1). Without the BLASTX option, HPC-BLAST has limited utility because it does not allow cross comparisons to be made between nucleotide and protein datasets.

Several other state-of-the-art software algorithms related to BLAST include PLAST and ScalaBLAST (Nguyen & Lavenier, 2009; Oehmen & Baxter, 2013). PLAST was designed to use processor cache memory, Single Instruction Multiple Data (SIMD) Supplemental Streaming SIMD Extensions 3 (SSE3) instruction set, multithreading, and a double-indexing scheme, but it can only be used within a single node containing multiple processor cores. Thus, PLAST is unable to take advantage of HPC systems with multiple nodes (Table 1). Similarly, BLAST+ supports the SSE3 instruction set as well as multithreading. ScalaBLAST was designed to run a large number of queries against either large or small databases based upon an MPI library. To improve upon BLAST, which is already highly efficient, ScalaBLAST uses a static load-balancing method centered on query sequence length. As a result, all the MPI cores will have the same query lengths and share a target database across virtually shared memory. This reduces the I/O bottleneck, takes advantage of the increased memory bandwidth, and has minimal latency. However, ScalaBLAST is limited because it supports only an older version of BLAST (BLAST revision 1.1.1.1 07/21/2006) and can suffer from memory management issues (Table 1).

In contrast, GPU-based BLAST software takes full advantage of large numbers of parallelized GPU cores (Vouzis & Sahinidis, 2011). However, running this type of parallelization on highly specialized GPUs has some inherent limitations. For example, the global memory of GPUs can limit the size of the dataset that can be analyzed, which means that running comparisons against large databases, such as the NCBI non-redundant database, might be problematic. Furthermore, GPU-BLAST is limited to only BLASTP (Table 1). One additional issue related to parallelization software for BLAST/BLAST+ is the utility of maintaining consistent *E*-values across the various implementations of BLAST/BLAST+ , which have become the sequence similarity standards for database searches. BLAST and BLAST+ *E*-values are calculated from three parameters: (1) the bit score, (2) the length of the query, and (3) the size of the database (Karlin & Altschul, 1990). Some parallelized implementations of BLAST, such as PLAST, do not maintain traditional *E*-values and bit scores and thus the results obtained cannot be compared directly to NCBI BLAST/BLAST+ outputs.

To overcome many of the obstacles outlined above, DCBLAST was designed to encourage the use of HPC computing systems to execute large sequence analysis jobs. Analysis of extremely large genomic and transcriptomic datasets for sequence similarities using NCBI BLAST/BLAST+ on a single node can be slow and delay downstream analyses. This is particularly true of transcriptome assembly files generated as descending-length, sorted transcript FASTA file outputs generated by algorithms such as Trinity (Haas et al., 2013), SOAPdenovo-Trans (Xie et al., 2014), and rnaSPAdes (Bankevich et al., 2012). Even if the user can split the query sequences into equal-sized query files, such as in SCBI_MapReduce (Guerrero-Fernández, Falgueras & Claros, 2013) or multiple random-sized query files, the job execution time for split BLAST will not be optimized due to length variations across the split files. Thus, the ability of DCBLAST to perform query balancing sets it apart from all other distributed approaches. Furthermore, DCBLAST is much easier to implement by a novice user than SCBI_MapReduce because it does not require users to directly access the Transmission Control Protocol/Internet Protocol (TCP/IP) to send jobs to specific computing nodes, a process which is not normally allowed in modern HPC systems that utilize job schedulers. SCBI_MapReduce users must also define the number of sequence subdivisions and the number of cores used for job processing. In contrast, DCBLAST fully automates these steps, which makes job execution easier to optimize than in SCBI_MapReduce, especially for extremely large datasets.

## Conclusion

We have used the Perl scripting language to develop the open-source software program DCBLAST as a powerful and simple implementation of BLAST to accelerate database searches. DCBLAST is an easy-to-use HPC computing wrapper for BLAST with a simple command-line interface that facilitates the processing of distributed sequence similarity searches for the novice user. DCBLAST dramatically reduces BLAST database search times for extremely large datasets by allowing distributed BLAST searches to be performed on HPC clusters. DCBLAST achieved this improved speed through simple, balanced, and automatic query splitting across the available cluster, grid, and cloud-based HPC resources, such as Amazon EC2 (Juve et al., 2009). This precise load balancing minimizes the run time for each available CPU, resulting in rapid job completion. Moreover, DCBLAST can be used with any type of DNA and protein sequence file, affording maximal flexibility to the user for *de novo* transcriptome or genome assemblies and more extensive genome-to-genome or multi-genome sequence analyses. Experiments with BLASTX suggest that the proposed DCBLAST will allow researchers to accelerate the execution of any program within the NCBI BLAST/BLAST+ suites as well as other sequence analysis programs such as HMMER (Eddy, 2011), BLAT (Kent, 2002), PLAST (Nguyen & Lavenier, 2009), and GPU-BLAST (Vouzis & Sahinidis, 2011). In summary, DCBLAST provides a simple, rapid, flexible, and easy method for the bioinformatics community to accelerate large-scale database sequence analysis tasks.

## Additional Information and Declarations

### Data Availability

The following information was supplied regarding data availability: DCBLAST: https://github.com/ascendo/DCBLAST, Operating system(s): Linux, Unix, Max OSX, Programming language: Perl 5.

## References

[ref-1] Altschul SF, Gish W, Miller W, Myers EW, Lipman DJ (1990). Basic local alignment search tool. Journal of Molecular Biology.

[ref-2] Bacon DF, Graham SL, Sharp OJ (1994). Compiler transformations for high-performance computing. ACM Computing Surveys.

[ref-3] Bankevich A, Nurk S, Antipov D, Gurevich AA, Dvorkin M, Kulikov AS, Lesin VM, Nikolenko SI, Pham S, Prjibelski AD, Pyshkin AV, Sirotkin AV, Vyahhi N, Tesler G, Alekseyev MA, Pevzner PA (2012). SPAdes: a new genome assembly algorithm and its applications to single-cell sequencing. Journal of Computational Biology.

[ref-4] Camacho C, Coulouris G, Avagyan V, Ma N, Papadopoulos J, Bealer K, Madden TL (2009). BLAST+: architecture and applications. BMC Bioinformatics.

[ref-5] Dagum L, Menon R (1998). OpenMP: an industry standard API for shared-memory programming. IEEE Computational Science and Engineering.

[ref-6] Dean J, Ghemawat S (2008). MapReduce: simplified data processing on large clusters. Communications of the ACM.

[ref-7] Eddy SR (2011). Accelerated profile HMM searches. PLOS Computational Biology.

[ref-8] Foster I, Berman F, Fox G, Hey A (2003). The grid: a new infrastructure for 21st century science. Grid computing: making the global infrastructure a reality.

[ref-9] Foster I, Zhao Y, Raicu I, Lu S (2008). Cloud computing and grid computing 360-degree compared.

[ref-10] Furlani JL (1991). Modules: providing a flexible user environment.

[ref-11] Furlani JL, Osel PW (1996). Abstract yourself with modules.

[ref-12] Gropp W, Lusk E (1997). A high-performance MPI implementation on a shared-memory vector supercomputer. Parallel Computing.

[ref-13] Guerrero-Fernández D, Falgueras J, Claros MG (2013). SCBI_MapReduce, a new ruby task-farm skeleton for automated parallelisation and distribution in chunks of sequences: the implementation of a boosted blast+. Computational Biology Journal.

[ref-14] Haas BJ, Papanicolaou A, Yassour M, Grabherr M, Blood PD, Bowden J, Couger MB, Eccles D, Li B, Lieber M, MacManes MD, Ott M, Orvis J, Pochet N, Strozzi F, Weeks N, Westerman R, William T, Dewey CN, Henschel R, LeDuc RD, Friedman N, Regev A (2013). *De novo* transcript sequence reconstruction from RNA-seq using the Trinity platform for reference generation and analysis. Nature Protocols.

[ref-15] Juve G, Deelman E, Vahi K, Mehta G, Berriman B, Berman BP, Maechling P (2009). Scientific workflow applications on Amazon EC2.

[ref-16] Karlin S, Altschul SF (1990). Methods for assessing the statistical significance of molecular sequence features by using general scoring schemes. Proceedings of the National Academy of Sciences of the United States of America.

[ref-17] Kent WJ (2002). BLAT–the BLAST-like alignment tool. Genome Research.

[ref-18] Krishnan A (2005). GridBLAST: a Globus-based high-throughput implementation of BLAST in a grid computing framework. Concurrency and Computation: Practice and Experience.

[ref-19] Lamesch P, Berardini TZ, Li D, Swarbreck D, Wilks C, Sasidharan R, Muller R, Dreher K, Alexander DL, Garcia-Hernandez M, Karthikeyan AS, Lee CH, Nelson WD, Ploetz L, Singh S, Wensel A, Huala E (2012). The Arabidopsis information resource (TAIR): improved gene annotation and new tools. Nucleic Acids Research.

[ref-20] Lin H, Ma X, Feng W, Samatova NF (2011). Coordinating computation and I/O in massively parallel sequence search. IEEE Transactions on Parallel and Distributed Systems.

[ref-21] Ling C, Benkrid K (2010). Design and implementation of a CUDA-compatible GPU-based core for gapped BLAST algorithm. Procedia Computer Science.

[ref-22] Matsunaga A, Tsugawa M, Fortes J (2008). CloudBLAST: combining MapReduce and virtualization on distributed resources for bioinformatics applications.

[ref-23] Nguyen VH, Lavenier D (2009). PLAST: parallel local alignment search tool for database comparison. BMC Bioinformatics.

[ref-24] Oehmen CS, Baxter DJ (2013). ScalaBLAST 2.0: rapid and robust BLAST calculations on multiprocessor systems. Bioinformatics.

[ref-25] Owens JD, Houston M, Luebke D, Green S, Stone JE, Phillips JC (2008). GPU computing. Proceedings of the IEEE.

[ref-26] Sato M, Boku T, Takahashi D (2003). OmniRPC: a grid RPC system for parallel programming in cluster and grid environment.

[ref-27] Sawyer SE, Rekepalli B, Horton MD, Brook RG (2015). HPC-BLAST: distributed BLAST for Xeon Phi clusters.

[ref-28] Shvachko K, Kuang H, Radia S, Chansler R (2010). The hadoop distributed file system.

[ref-29] The UniProt Consortium (2013). Update on activities at the Universal Protein Resource (UniProt) in 2013. Nucleic Acids Research.

[ref-30] Vouzis PD, Sahinidis NV (2011). GPU-BLAST: using graphics processors to accelerate protein sequence alignment. Bioinformatics.

[ref-31] Xie Y, Wu G, Tang J, Luo R, Patterson J, Liu S, Huang W, He G, Gu S, Li S, Zhou X, Lam T-W, Li Y, Xu X, Wong GK-S, Wang J (2014). SOAPdenovo-Trans: *de novo* transcriptome assembly with short RNA-Seq reads. Bioinformatics.

